# How Do Instant Messages Reduce Psychological Withdrawal Behaviors?—Mediation of Engagement and Moderation of Self-Control

**DOI:** 10.3390/ijerph18062983

**Published:** 2021-03-14

**Authors:** Xia Jiang, Jing Du, Tianfei Yang, Yujing Liu

**Affiliations:** School of Economics and Management School, Wuhan University, Wuhan 430072, China; jiangxia@whu.edu.cn (X.J.); jdu@whu.edu.cn (J.D.); liuyujing@whu.edu.cn (Y.L.)

**Keywords:** instant message, engagement, self-control, psychological withdrawal, COR

## Abstract

Enabling people to send and receive short text-based messages in real-time, instant messaging (IM) is a communication technology that allows instantaneous information exchanges. The development of technology makes IM communication widely adopted in the workplace, which brings a series of changes for modern contemporary working life. Based on the conservation of resource theory (COR), this paper explores the mechanism of workplace IM communication on employees’ psychological withdrawal, and investigates the mediating role of work engagement in the relationship and the moderating role of self-control. Using the experience sampling method (ESM), a 10-consecutive workdays daily study was conducted among 66 employees. By data analysis of 632 observations using SPSS and HLM, results found that: (1) IM demands had a positive relation with emotion and cognitive engagement. (2) Emotion and cognitive engagement were negatively correlated with psychological withdrawal. (3) Emotion and cognitive engagement mediated the relations of IM demands and psychological withdrawal. (4) Self-control moderated the relationship between emotional engagement and psychological withdrawal.

## 1. Introduction

Nowadays, mobile communication technology has become a crucial part in the highly connected working context [1]. In light of instant connections and enhanced communication, modern organizations have adopted a several of technologies to facilitate organizational activities. Smartphones serves as implementation tools that can be widely used for many work-related issues, like online meetings, schedule management and document sharing [2]. However, the consequence of instant message (IM) usage on employees’ psychological well-being is still controversial [3]. Several studies have provided empirical evidence that excess instant messages are related to feelings of strain [4,5,6]. Brown, et al. [7] reported that many employees spend at least 20% of their work time handling messages, and this amount is expected to increase over time. The request for immediate response needs employees to manage the flow of instant messages, leading to interruptions and decreased organizational productivity [8,9]. Despite the disruptive impact of mobile communication technology, there is no denying that IM facilitates work communication and enhances team performance for the most part [10]. Flexible communication with IM may help bridging resources that in turn can be used for personal gains [11]. It is a significant omission that in the context of IM demand we have yet to discuss how such task-support process influences employees’ daily behavior via work engagement.

The purpose of this study is to add new knowledge to the existing literature on organizational behaviors by examining how IM demands affect work engagement which further affects employees’ psychological withdrawal behaviors. The present study is the first of its kind to explore the IM demands, work engagement, self-control, and psychological withdrawal behavior altogether. There is no need to illustrate how important instant messages are in our modern work life. The main viewpoint about instant communication usually takes instant messages as work interruptions, talking of negative effects on certain work outcomes. However, in our study, we argued that from the conservation of resource theory (COR) perspective, instant messages carry a lot of work-related information and indication. Employees may get resources and emotional recovery from instant interactions with others [11]. Using COR, we developed a model to explain how instant messages help work behaviors. The timeliness of IM facilitates “anytime” communication in the workplace. The main advantage of IM was convenient personal interaction, and employees could quickly provide feedback on questions and ongoing work tasks [12]. When the employees get information, messages, and updates, they can do better in their jobs. While they read and respond to these messages, there are some transient breaks from intense work tasks, which could be a time-to-time recovery for the employees. In that case, employees could both get resources and recovery for work by receiving instant messages. Thus, they are more likely and capable of engaging in work tasks. The more support they get from instant messages, the more they devote themselves to work, and the less they perform psychological withdrawal behaviors. 

**Figure 1:**
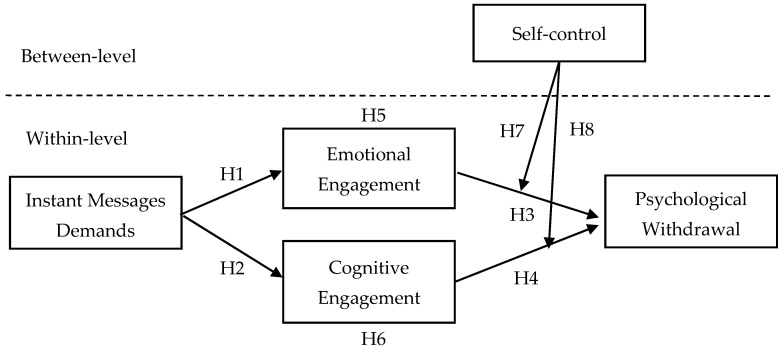
Theoretical model.

Besides, we look inside the potential boundary conditions that may influence the relationship in our model. In day-to-day work life, people need to manage various instant messages. The personal trait plays a role and differs the relationship. Self-control indicates one’s ability to regulate immediate emotions, impulses, thoughts, and feelings. Individuals with high trait self-control can do better on behavior control [13,14]. Merge the model, our study clarifies how the instant messages flourish the day-to-day work and when the individual trait influences the effect (see Figure 1).

## 2. Theory Framework and Hypotheses 

### 2.1. Instant Message Demands 

IM refers to the instant transmission of voice messages, files, pictures, video, and other messages through online chat technology [15]. Nowadays, IM is widely used in workplaces to enhance communication within organizations [16,17,18,19]. Instant messaging tools such as WeChat, DingTalk, and Tencent QQ that Chinese people use daily are increasingly used for professional purposes to help corporate personnel work together, manage information and maintain relationships [16,20]. IM demands mean that employees expect every instant message to be read and responded to more or less when it arrives. Previous studies have found that employees believed that they must read and respond to instant messages as soon as possible, otherwise they would worry about losing important information [21]. In addition, employees who initiate instant messages also hope to get a quick response from the other party. This sense of urgency has formed a consensus that both parties want to communicate faster and more efficiently [22]. In fact, every message will be read and responded to more or less when it arrives. This expectation has become the norm for workplace communication [23].

### 2.2. Emotional and Cognitive Engagement 

Kahn defined work engagement as employees controlling themselves to integrate themselves with job roles [24], accepting the goals and values of the organization, and doing their best to help the organization become better. Work engagement is a kind of psychological feeling of employees, that is, a state in which employees feel that their energy can be focused in the process of work. When employees are fully engaged in job tasks, whether alone or with others, they show high engagement. They will remain cognitively alert, focused, and emotionally connected with their work and co-workers [24].

Emotional and cognitive engagement are the two aspects of work engagement, which is a multidimensional motivational concept [25]. Individuals exhibit work engagement through investing their emotional and cognitive energies into role performance. To be specific, emotional engagement means employees have an empathetic connection to others, and they express excitement in their work that can reflect the level of pleasantness and activation [26]. Cognitive engagement incorporates being aware, actively thinking and tracking information, questioning and displaying genuine curiosity, and being focused and absorbed in working [27]. In terms of employees’ emotional and cognitive energies can be allocated in various work according to the definition and state about their work [28].

### 2.3. Self-Control

Tangney et al. [14] pointed out that self-control is the ability to suppress inner irrational impulses and not turn impulses into actions. Duckworth [29] believes that self-control is the ability of individuals to restrain themselves for long-term goals and to suppress emotional and behavioral impulses more effectively. The core concept is the ability to transcend or change a person’s inner reaction, as well as to interrupt bad behavior tendencies and avoid taking actions against them [14]. Previous studies have shown that there are significant individual differences in people’s self-control ability. Some people are more capable than others to manage their own life, emotions, language, diet, consumption, etc. This is a key individual difference that explains why some people are more capable than others to control impulses, maintain attention, and act positively, and put resources into long-term goals pursuit activities [13,30,31].

### 2.4. Psychological Withdrawal Behavior

Lehman and Simpson [32] described psychological withdrawal behaviors as “*a series of neglect behaviors at work, negatively related to work performance*”. As a kind of work withdrawal behavior, psychological withdrawal behavior does not need to invest a lot of time, energy, and other resources. It mainly refers to a series of intentional negative work behaviors taken by employees to avoid work or weaken the social and psychological connection between themselves and their organization [33]. Psychological withdrawal behaviors represent psychological withdrawal from the work situation, including wanting to be absent, daydreaming, doing personal tasks at work, chatting excessively with colleagues, putting little energy into work, and letting others do the tasks [34]. As a negative workplace behavior, if it is not sufficiently recognized and managed by managers, less serious psychological withdrawal behaviors may cause employees to perform harsh exit behaviors, such as turnover intentions [34] and actions, which may not only cause economic losses to the organization but also damage organizational norms and have a negative demonstration effect among employees [34,35]. Therefore, it is very important to explore how to reduce psychological withdrawal. Previous research emphasized that researchers paid insufficient attention to withdrawal behavior and called on scholars to conduct more related research [34,36,37].

### 2.5. Conservation of Resource Theory

Conservation of resource theory (COR, Hobfoll [38,39]) proposes that individuals will strive to protect, obtain and construct valuable resources for themselves, and try to avoid the consumption and loss of valuable resources. Hobfoll et al. [40] revised the COR theory, and finally clarified the basic point of the COR theory as a basic tenet. Individuals tend to strive to acquire, maintain, cultivate and protect their cherished resources. Individuals will use their existing resources as much as possible to adapt to the environment. Successful adaptation will generate new resources so that they can have more resources to withstand the pressure they may face in the future. Instant messages are almost inevitable in the workplace, so it is a smarter choice for employees to actively accept and respond to instant messages in the work environment. By receiving and responding to instant messages, not only can it bring work-related information and resources to employees, but it can also maintain and strengthen the work and social relations of employees to a certain extent. In other words, employees can promote the preservation of valuable resources by actively constructing and protecting their resource reserves.

### 2.6. Relationships among Study Variables

A core theme of IM research is its negative effect associated with work interruptions [41]. IM promotes the wide sharing of news, information, and updates “at any time” among employees of the organization [30,42]. For the recipient, checking, reading and responding to instant messages, and then refocusing their attention back to work requires a continuous cognitive effort [30]. Previous studies [43] suggest that reading and replying to electronic information will distract attention, lead to work interruption and require employees to transfer resources from other activities. There is an implicit assumption here. The instant messages received will divert attention and interrupt the work at hand. Is that all about? It is no doubt that IM is all around our life. Modern work is inevitably affected. Handling IM is a part of the job. We suggest that receiving instant messages is essentially a form of communication, and whether it is interrupted depends on the content of the message. Previous studies seeing IM as interruption overlooked the possible impact of the content of the news. The messages received by individuals may seek help and cooperation, and may also provide support and cooperation. As a carrier of resources, news, and support, instant information provides employees with feedback and prompts to advance tasks, so that employees can obtain practical and emotional support, and increase employees’ resources for work. Therefore, we propose the following assumptions.

**Hypothesis** **1.** *IM demands will have a positive relation with emotional engagement.*

**Hypothesis** **2.** *IM demands will have a positive relation with cognitive engagement.*

Previous studies have shown that there is a significant relationship between work engagement and psychological withdrawal behavior [44,45]. Zhong and Liu [46] showed that the higher the employees’ work engagement, the less their non-work behaviors. When employees have a high level of work engagement, they often devote themselves to work, overcome difficulties, persevere, and never stop until they reach their goals. In addition, employees with a high level of work engagement will agree with the company and their work, have very clear goals, so they are very proactive in their work. Based on the above research, work engagement can improve employees’ work performance and reduce psychological withdrawal behavior. Therefore, this research proposes the following hypotheses.

**Hypothesis** **3.** *Emotional engagement will have a negative relation with psychological withdrawal.*

**Hypothesis** **4.** *Cognitive engagement will have a negative relation with psychological withdrawal.*

According to COR, individuals strive to acquire, maintain, nurture and protect the resources. This is the core of the concept. COR emphasizes the need for people’s motivation to acquire, maintain, cultivate and protect their resources comes from the basic needs of human beings as a species to adapt to the environment and survive out of the stiff competition. IM communication with colleagues will bring work-related information resources. At the same time, it is a supplement to interpersonal resources and social resources, which may increase work input. Through work engagement, employees continue to accumulate work-related resources, and they are likely to find that work becomes more interesting and attractive, so that they are not afraid of difficulties and reduce psychological withdrawal behavior. In other words, high work engagement may reduce psychological withdrawal behavior. We propose the following hypotheses accordingly.

**Hypothesis** **5.** *Emotional engagement will mediate the relations of IM demands and psychological withdrawal.*

**Hypothesis** **6.** *Cognitive engagement will mediate the relations of IM demands and psychological withdrawal.*

Previous studies have shown that, compared with employees with weak self-control, employees with strong self-control perform better [47], are more trustworthy, and act more responsible [48]. Empirical evidence shows that people with high levels of self-control perform better in all fields [48]. Those who have a higher level of self-control have the appropriate ability to maintain attention, control impulse, switch behavior, and allocate transformational resources to goal pursuit activities [30,49]. Hypothesis 3 suggests that the more emotional and cognitive engagement, the less psychological withdrawal of employees. For individuals with stronger trait self-control, they have more resources [13,50] to maintain attention, control impulse, and reduce withdrawal behavior. Individuals with high self-control can make their behaviors, thoughts, and emotions meet social expectations, and can firmly pursue long-term goals. Therefore, we hypothesize that when self-control is high, the negative correlation between emotional intervention and psychological withdrawal will be stronger.

**Hypothesis** **7.** *Self-control will positively moderate the relationship between emotional engagement and psychological withdrawal.*

**Hypothesis** **8.** *Self-control will positively moderate the relationship between cognitive engagement and psychological withdrawal.*

## 3. Method 

### 3.1. Participants and Procedure

Our sampling strategy includes recruiting full-time employees from a wide range of jobs, which can increase the generalizability of our research. Participants were selected who were (1) employed in a full-time job, (2) worked in the Beijing Time Zone of China, and (3) typically started their workday between 12:00 a.m. to 1:00 p.m. and ended between 5:30 p.m. to 7:00 p.m. We published recruitment information on the Internet, and through personal contact. In the end, a total of 66 participants passed these criteria. Participants were awarded a small honorarium equivalent to $30.86 if they completed the full 10 day survey. 

Data collection for this study lasted three weeks. In the first week, we conducted an initial survey of participants, investigating their demographic information and assessing self-control traits. In the second and third weeks, we started sending electronic questionnaires with a link to two different surveys for 10 consecutive workdays. The first daily survey was sent at lunchtime, which contained the measures of IM demands, emotional, and cognitive engagement. The second daily survey was conducted at the end of the workday, assessed psychological withdrawal.

We used an empirical experience sampling methodology (ESM) [30,51] to collect data, which enabled us to explore the within-person dynamics processes [30] implied by our framework. After data collection was complete, we eliminated missing responses and unmatched data. Our final sample comprised 632 daily observations from 66 employees in China, the effective response rate was 95.76%. The sample was comprised of 18 (or 27.3%) men and 48 women (or 72.7%). The average age of participants was 30.56 years old (SD = 4.81), average work experience of participants was 7.71 years (SD = 5.28). Of the respondents, 28.80% were single and 71.20% were married. Participants occupied a variety of positions in their organizations such as HR service agent, financial analyst, structural engineer, teacher, administrators, web designer, etc. And they also came from various walks of life, such as real estate, automobile manufacturing, legal profession, service industry, sales industry, and others.

### 3.2. Measures 

#### 3.2.1. Instant Messages Demands 

Since the research on IM is very new and the existing scales are not very mature, we quoted and adapted the previous scales. We measure instant message demands by adapting items from Dabbish and Kraut [52] and Rosen, et al. [30]. The four items are as follow: “I received a lot of instant messages this morning”, “I read a lot of instant messages…”, “I replied to a lot of instant messages…”, “I handled a lot of instant messages…”. Average coefficient alpha across the 10 days was 0.948.

#### 3.2.2. Emotional and Cognitive Engagement

We measured emotional and cognitive engagement using 12 items adapted from Byrne et al. [27] and Rich et al. [25] indicate day-level job engagement. 

Emotional engagement. e.g., “Today at work, I am enthusiastic in my job”,” …I feel energetic at my job”,” … I am interested in my job”, “ …I am proud of my job”,” …I feel positive about my job”,” …I am excited about my job”. Average coefficient alpha across the 10 days was 0.954.

Cognitive engagement. e.g., “Today at work, my mind is focused on my job”, “…I pay a lot of attention to my job”, “…I concentrate on my job”, “…I focus a great deal of attention on my job”, “…I am absorbed in my job” and “…I devote a lot of attention to my job”. Average coefficient alpha across the 10 days was 0.962.

#### 3.2.3. Self-Control

We assessed self-control using a 13-item scale developed by Tangney, et al. [14]. e.g., “I am good at resisting temptation”, “I have a hard time breaking bad habits”, “I am lazy”, “I say inappropriate things”, “I do certain things that are bad for me, if they are fun”, “I refuse things that are bad for me”, “People would say that I have iron self-discipline”, “I am able to work effectively toward long-term goals”. Average coefficient alpha was 0.752.

#### 3.2.4. Psychological Withdrawal Behaviors

We measured daily psychological withdrawal behaviors by adapting 7-items from Lehman and Simpson [32]. e.g., “Today at work, I spent work time on personal matters”, “…Put less effort into job than should have”, “…Daydreaming”, “…Left work station for unnecessary reasons’’, and “…Thoughts of being absent.” Average coefficient alpha across the 10 days was 0.912.

All items are self-evaluated by employees on a Likert 5-point scale (“1” to “5” range from “very disagree” to “very agree”). Coefficients α are all greater than 0.7, indicating good internal consistency.

### 3.3. Analysis

Since we used ESM, our data was cross-level at the within-level and between-level. According to the recommendations by Hofmann et al. [53], we group mean centered our continuous within-level variables and grand-mean centered our between-level variable. At the level 1 hypotheses test, we chose IBM SPSS Statistics 20.0 (International Business Machines Corporation, Armonk, NY, USA) to examine the main effect (H1 & H2, H3 & H4) and mediation (H5 & H6). Indirect effects were tested using procedures in accordance with recommendations by Preache et al. [54]. Confidence intervals (CIs) were constructed using parametric bootstrap procedures [55].

In consideration of the nested structure of our data (daily observations nested within individuals), we chose HLM software version 6 (Scientific Software International Inc., Lincoln Wood, IL, USA) to test our cross-level moderating effect (H7 & H8). In accordance with the recommendations by Wang et al. [55], all within-level slopes were modeled as random. Self-control was modeled as a cross-level moderator predicting the slope between emotional engagement and cognitive engagement with psychological withdrawal (moderating effect).

Following the recommendations by Preacher et al. [56], we conducted simple slope tests to verify the moderating effect of emotional engagement and cognitive engagement with psychological withdrawal, and draw the plots.

## 4. Data Analysis and Results

### 4.1. Descriptive Data Analysis

Means, SDs, and intercorrelations among study variables are reported in Table 1. Before we started hypotheses testing, we ducted a confirmatory factor analysis to verify the distinctiveness of our study variables. Instant messages demand, cognitive engagement, emotional engagement, and psychological withdrawal were included at the within-level. And the model displayed acceptable fit well (χ^2^ = 107(29), *p* < 0.0001, comparative fit index [CFI] = 0.984, Tucker-Lewis index [TLI] = 0.975, root mean square error of approximation [RMSEA] = 0.0649, CI = [0.0519, 0.0783]), which supported the distinctiveness of our study variables.

### 4.2. Results of Hypothesis Testing

**Figure 2:**
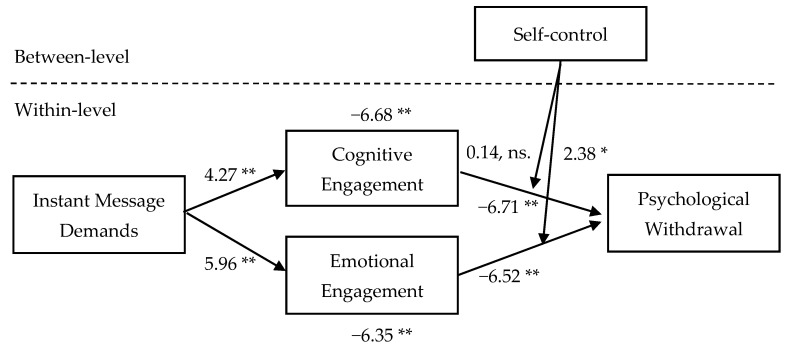
Multilevel path analysis. Note. * *p* < 0.05. ** *p* < 0.01.

**Figure 3:**
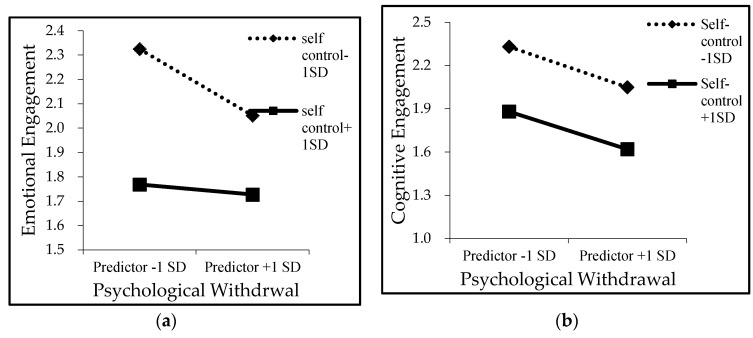
(**a**) Cross-level moderating effect of self-control on the relationship between emotional engagement and psychological withdrawal. (**b**) Cross-level moderating effect of self-control on the relationship between cognitive engagement and psychological withdrawal.

Results of our multilevel path analysis are shown in Figure 2 and Table 2. In consistent with our hypotheses 1 (B = 0.16, *p* < 0.01) and 2 (B = 0.11, *p* < 0.01), IM demands were positively related to emotional engagement and cognitive engagement, indicating that more IM demands would increase employees’ emotional and cognitive engagement. In hypotheses 3 and 4 we predicted emotional engagement and cognitive engagement would have a negative relation with psychological withdrawal. As Figure 2 shows, we found both negative relations, emotional engagement with psychological withdrawal (B = −0.31, *p* < 0.01), and cognitive engagement with psychological withdrawal (B = −0.32, *p* < 0.01), are significant. Hypotheses 5 and 6 predicted that emotional engagement and cognitive engagement would mediate the relations of IM demands psychological withdrawal. We found support for them, as the CIs for the indirect effect of IM demands and psychological withdrawal through emotional engagement (B = −0.049, 95% CI [−0.075, −0.026]), and cognitive engagement (B = −0.037, 95% CI [−0.060, −0.016]), shown in Table 3. Hypothesis 7 and 8 predicted that self-control would moderate the relationship between emotional engagement and cognitive engagement with psychological withdrawal. As Figure 2. shows, self-control had significant effects on the emotional engagement-psychological withdrawal slope (B = 0.238, *p* <0.01), and had no significant effects on the cognitive engagement-psychological withdrawal slope (B = 0.021, ns.). In support of these results, as shown in Figure 3a,b, emotional engagement had stronger relations with psychological withdrawal when self-control was low than was high, and the relationship between cognitive engagement and psychological withdrawal was not significantly influenced by self-control. Thus, hypothesis 7 was supported, 8 was not.

## 5. Discussion

To summarize, based on the COR theory, this study illustrated how IM contributed to work behaviors. We used ESM, conducting a 10-consecutive workdays survey. It turned out that, IM demands were positively associated with emotional engagement and cognitive engagement. Cognitive engagement and emotion engagement had a negative relation with psychological withdrawal, mediating the relations of IM demands and psychological withdrawal. These results indicated that IM played an important role in daily work, for building social communication and relation [57], requiring rapid responsiveness, and supporting work engagement [58]. In consideration of personal traits, self-control was taken into account as a conditional factor, moderating the relationship between emotional engagement and psychological withdrawal, while cognitive engagement was not supported. Employees with high self-control are more capable of managing impulses and actions. Thus, with the ability of self-control, employees could reduce more psychological withdrawal when in emotional engagement.

### 5.1. Theoretical and Practical Contributions

IM now has been widely used in the workplace and changes some work behaviors and even traditions. With a fast network, this kind of near-synchronous interactional communication contributes to greater efficiency for organizational immediate interchange. Our findings showed important meanings both for IM research and IM use in workplace practice.

First of all, this paper constructs a framework in arguing with COR theory to explain how IM flourish work behaviors and work performance, which challenges the point of seeing instant messages as interruptions for work. This expands the research on the utility of IM. Secondly, we take the point of view that IM should be considered from the message itself. Receiving messages from others is not just about receiving behaviors. Through this near-synchronization communication technology, employees talk more freely and effectively, which enables real-time information exchanges and increases responsiveness. Hence, employees with higher energy and resource for engaging in work. That is providing a thought about how the IM influences the workplace. Thirdly, we drawl self-control as the boundary condition. As we pay close attention to the daily effect of IM, personal traits could be the main consideration for conditional moderator. We combine the daily change with individual difference, to look deep inside of the mechanism about IM. This should be a complement for IM study.

There are some practical contributions to our research findings that require managers’ attention. IM is indispensable for daily work in modern society. Although its use can be conceded as a kind of distraction [59], such communication technology is still useful by its interactivity and convenience to most employees. Recognizing the positive effects of IM demands fit, managers need to concern their employees’ personal demands to identify problems in subsequent engagement and behaviors. To be specific, organizations should develop rules and strategies to help satisfy employees’ IM demands, ensuring they avoid sending and receiving too much needless instant message. Employees will work more effectively and efficiently with such favorable usage experiences. Also, employees should exercise and take care of their self-control ability consciously. When work-related communication issues are perceived as barriers to motivate employees’ work engagement, sufficient self-control should be encouraged as more effective alternative resources to reduce their psychological withdrawal behavior.

### 5.2. Limitations and Suggestions for Future Research

A few limitations in our study should be noted. First of all, we used a single survey to collect self-reported perception data. Future research could rely on multi-source data which are measured from different participants or quantitative data about instant message demands. Combining multi-source data and quantitative data will provide more convincing evidence for the research results. On the side, the participants of our study are all Chinese employees, which could limit the generalizability of our findings. Future research should consider that expanding the sample characteristic based on different cultural dimensions, to determine whether the regional culture may play a role concerning the influence of instant message demands. Finally, we only examined the moderating effect of self-control. Although employees’ personality traits were proved to be related to psychological withdrawal behavior [60], variables from other levels, such as team climate and leadership style, potentially also may bring influences to the relationship between engagement and psychological withdrawal behaviors. Therefore, we encourage researchers to extend the boundary conditions further, which will open up another perspective for the investigations of instant messages demands in organizational practices.

## 6. Conclusions

With the rapid development of network technology and communication technology, new technology not only transformed social production structure but also the way people live. IM originally intended to allow home internet users to have conversations with family and friends [61]. However, it is now been not only at home but also at the workplace. Real-time communication makes things easier for managers and employees, helping them with information exchange, process feedback, and emotional connection, almost anywhere and anytime [8,62]. If it has made organizational communications more effective, still in discussion. Our study provided empirical evidence that IM demands would positively correlate to emotional engagement and cognitive engagement, which then cut down psychological withdrawal. And self-control as the individual factor would moderate the relationship between engagement and psychological withdrawal. To use IM more efficiently, there are calling for deep and intensive explorations.

## Figures and Tables

**Table 1:** Means, SDs, and intercorrelations among study variables.

Variable	Mean	SD	1	2	3	4
*Within*						
1. Instant Message Demand	3.00	0.89	1	0.167 **	0.233 **	−0.134 **
2. Cognitive engagement	3.73	0.60	0.504 **	1	0.654 **	−0.489 **
3. Emotional engagement	3.54	0.61	0.550 **	0.895 **	1	−0.486 **
4. Psychological withdrawal	1.96	0.65	0.121	−0.057	−0.098	1
*Between*						
5. Self-control	3.43	0.40	0.102	0.234	0.334 **	−0.326 **

Note. N at Level 1 = 632, N at Level 2 = 66. SD = standard deviation. Variables 1–4 are within-individual (Level 1) variables. Their means and SDs are based on between-person scores. Intercorrelations above the diagonal are based on within-individual scores; intercorrelations below the diagonal are based on between-individual scores. Self-control is a between-individual variable. The intercorrelations of self-control with variables 1–4 are based on between-individual scores (i.e., we aggregated variables 1–4 at the individual-level). ** *p* < 0.01.

**Table 2:** Relations between IM demands, emotional engagement, cognitive engagement and psychological withdrawal.

Variable	Cognitive Engagement			Emotional Engagement			Psychological Withdrawal		
Index	B	SE	*t*	B	SE	*t*	B	SE	*t*
Intercept	3.39	0.08	40.59 **	3.07	0.08	36.65 **	4.27	0.15	29.08 **
*Within-level*									
IM demands	0.11	0.03	4.27 **	0.16	0.03	5.96 **			
Cognitive engagement							−0.32	0.05	−6.71 **
Emotional engagement							−0.31	0.05	−6.52 **

Note. N = 632 observations nested within 66 individuals. Within-level predictors were group mean centered. Unstandardized coefficients are reported. B = effect. ** *p* < 0.01.

**Table 3:** Mediation effect of emotional engagement and cognitive engagement.

Psychological Withdrawal	B	Boot SE	95%CI	
			LLCI	ULCI
Direct effect	−0.013	0.025	−0.063	0.037
Indirect effect(s)				
TOTAL	−0.085	0.019	−0.124	−0.046
Emotional engagement	−0.049	0.013	−0.075	−0.026
Cognitive engagement	−0.037	0.011	−0.060	−0.016

Note. Level of confidence for all confidence intervals in output: 95. Number of bootstrap samples for percentile bootstrap confidence intervals: 5000. B = effect.
